# Development of a Healthy Lifestyle Mobile App for Overweight Pregnant Women: Qualitative Study

**DOI:** 10.2196/mhealth.9718

**Published:** 2018-04-23

**Authors:** Ying Lau, Ling Jie Cheng, Claudia Chi, Cammy Tsai, Kai Wen Ong, Sarah Su Tin Ho-Lim, Wei Wang, Kian-Lee Tan

**Affiliations:** ^1^ Alice Lee Centre for Nursing Studies Yong Loo Lin School of Medicine National University of Singapore Singapore Singapore; ^2^ Department of Nursing, Khoo Teck Puat Hospital Yishun Health Campus National Healthcare Group Singapore Singapore; ^3^ Department of Obstetrics & Gynecology National University Hospital Singapore Singapore; ^4^ Department of Rehabilitation National University Hospital Singapore Singapore; ^5^ Department of Dietetics National University Hospital Singapore Singapore; ^6^ Department of Computer Science National University of Singapore Singapore Singapore

**Keywords:** mobile apps, overweight, obesity

## Abstract

**Background:**

Mobile apps are becoming an increasingly ubiquitous platform for delivery of health behavior interventions among overweight and obese perinatal women. However, only a few methodological guidelines on integrating theory, evidence, and qualitative research for their designs are available.

**Objective:**

The aim of this study was to develop a theory-based, evidence-driven, and user-centered healthy lifestyle app targeting overweight and obese multiethnic pregnant women.

**Methods:**

This paper illustrates how intervention development may be enriched with theoretical basis, systematic review, and qualitative study. An individual face-to-face interview was performed to incorporate the user’s involvement in the design. These interviews were audiotaped and transcribed. Thematic analysis technique was used for emerging themes.

**Results:**

Integrated concepts of social cognitive theory of self-regulation, self-regulation model, and strength model of self-control were selected as bases of the intervention. Evidence from our systematic review and meta-analysis provided the strongest evidence for the development of intervention. We invited 16 obese or overweight pregnant women to participate in a semistructured interview *.* The following key themes emerged: content, platform, interactivity, format, and functionality. Apps are a favorable technology platform for healthy diet advice, appropriate physical exercise, and weight management because they are user-friendly and convenient. The app used in this study contains culture-specific, pregnancy-related, and credible contents, including educational, professional and peer support, and self-monitoring domains. The design should include aesthetic appeal, visualized features, and interactive multimedia.

**Conclusions:**

A 3-step process integrating theoretical basis, evidence from systematic review, and research findings from target users can be considered a guide for future app development.

## Introduction

### Background

With obesity as a worldwide epidemic [1], perinatal overweightness and obesity have been widely considered [1]. Approximately 50% of women experience excessive gestational weight gain (GWG) [2], which shows a consistent relation to postpartum weight retention [3] and a substantially increased risk of being obese. Perinatal overweightness and obesity are linked to adverse maternal and infant outcomes, including obstetric, intrapartum complications, instrumental delivery, babies who are large for their gestational age, and macrosomia [2,4,5]. Unhealthy lifestyle patterns are critical factors influencing perinatal overweightness and obesity [6]. Behavioral change depends on the discontinuation of an unhealthy lifestyle and the formation of a new healthy lifestyle [7]. Pregnancy is a crucial stage to remain healthy for the sake of pregnant women and their unborn baby [8]. Healthy eating during pregnancy is critical, and pregnant women require 2000 kcal/day [9]. To obtain a balanced diet, pregnant women should eat a variety of food, including fruits, vegetables, rice, meat, and milk and its alternatives [9]. With regard to physical activity, pregnant women should walk 10,000 steps a day (4-5 miles, depending on stride length) or do a minimum of 30-min moderate physical activity for 5 to 7 days a week [10]. The Institute of Medicine guidelines recommend a total weight gain during pregnancy based on the prepregnancy body mass index (BMI) as follows: normal, 11.5 to 16 kg; overweight, 7 to 11.5 kg; and obese, 5 to 9 kg [11]. Consequently, new and effective lifestyle interventions that promote healthy outcomes are necessary.

Mobile apps create new opportunities to set behavioral goals, provide healthy lifestyle counseling, and facilitate self-monitoring of pregnant women’s goal-directed behavior [12]. Apps have become increasingly relevant to health care. Apps have been successfully integrated into interventions that target diet, physical activity, and weight management in overweight and obese individuals [13]. Apps also use a tracking system to improve adherence by automatic alert or notification or graphic progress by monitoring devices for reminders and regular interactions. The advantages of using apps include cost-effectiveness, accessibility, and timely delivery to multiple regions and various populations [13,14]. Perinatal women actively use apps to search for pregnancy health–related information, discuss issues with peers, and seek advice from professionals to guide their pregnancy decision making [15,16]. Nonetheless, the market for health care apps is considerably fragmented because many of them are designed for highly specific contexts, and they lack theoretical content. A systematic review on quality assessment for apps [17] is varied. Results showed that only 10 studies from 606 articles satisfy the inclusion criteria. According to the quality criteria, the mean score is 5.05 out of 8 [17]. Thus, the development of a quality and evidence-based app for overweight or obese perinatal women is necessary.

Theoretical-based intervention helps guide intervention designers in identifying theoretical constructs to target in an intervention to elicit behavioral change [18]. In addition, theoretical foundation provides guidance on mobile health behavior intervention development [19]. The Template for Intervention Description and Replication (TIDieR) guideline [20] recommended the use of theoretical frameworks in designing interventions. More importantly, theory-informed development facilitates the functionality of an intervention [21]. Evidence from systematic review and meta-analysis is used as a basis to develop recommendations for mobile app development [22]. Systematic review is the reference standard in synthesizing evidence in health care [23]. Moreover, a meta-analytic approach is considered the strongest evidence because of its methodological rigor [24]. User-centered design is a well-established approach to develop a mobile app. This design is strategically important because of its insights on users and their context of use [25]. Advantages of user-based design include the promotion of autonomy, competence, positive emotional experience, and sense of relatedness for users [26]. User-based design focuses on target audience through an iterative design process that engages users in conceptualization, design, and development of an app [27]. User involvement increases appeal and user-friendliness [28]. Target population can select tailored information about their preferred form, which is essential to maximize the acceptability and effectiveness of interventions [26]. User-centered development method can assist in understanding the preference of potential users for content, platform, and format, thereby resulting in a remarkably effective program.

### Objectives

With potential benefits of low cost, high accessibility, and good adherence, this study aims to develop a mobile app (mHELP) for a healthy lifestyle program among overweight or obese multiethnic pregnant women using a theory-based, evidence-driven, and user-centered approach.

## Methods

With regard to mHELP development, we used a 3-step process by integrating theoretical basis, evidence from our systematic review, and research findings from our target users.

### Step 1: Theory-Informed Development

**Figure 1:**
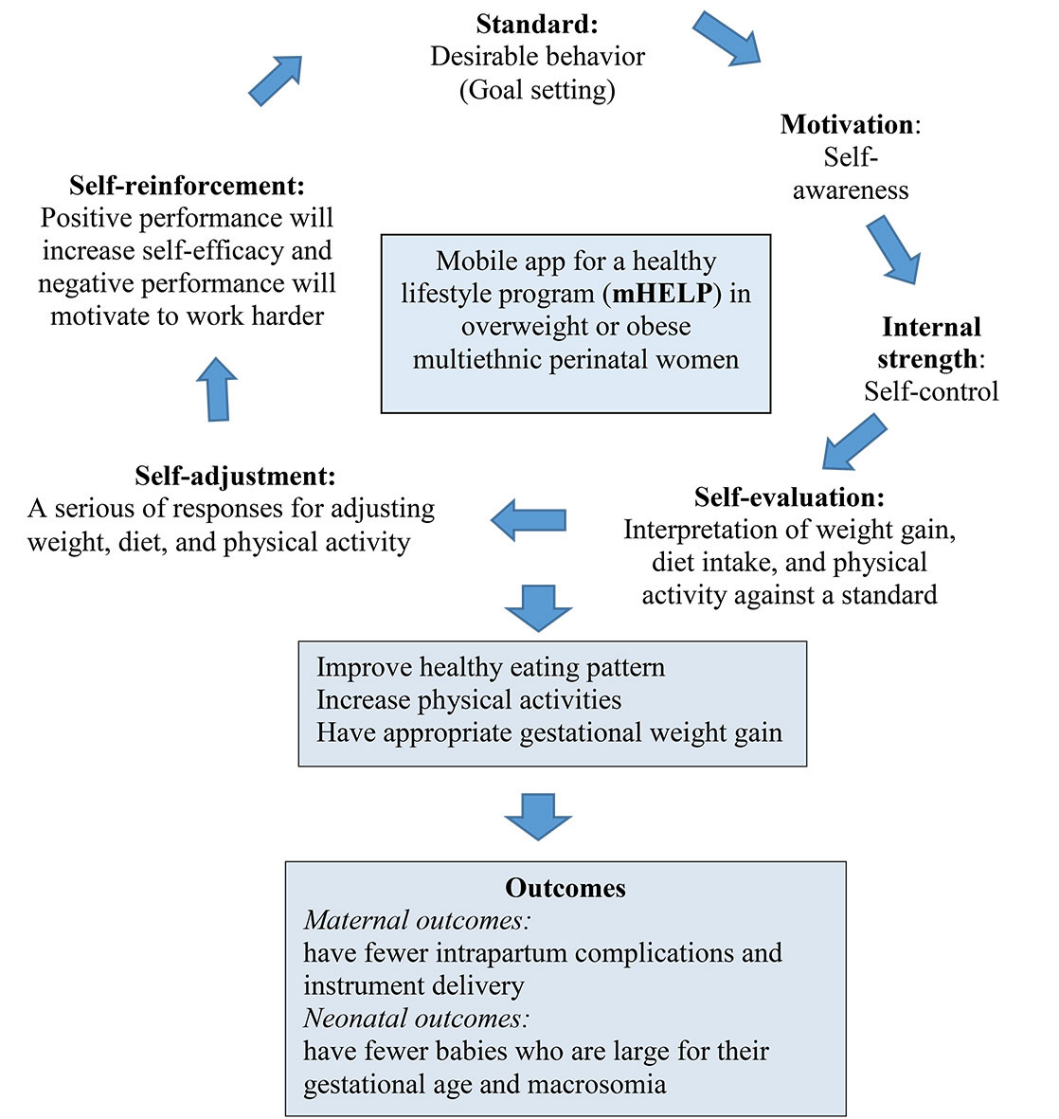
Conceptual framework.

Intervention development by using a theoretical basis can substantially improve health behavior [18]. Hence, mHELP development was based on the integrated concepts of social cognitive theory of self-regulation [29], self-regulation model [30], and strength model of self-control [31]. The social cognitive theory of self-regulation emphasizes the major self-regulative mechanism through the 3 principal subfunctions, including self-monitoring of individual behavior (its determinants and its effects), evaluation of individual behavior in relation to personal standards and environmental circumstances, and affective self-reaction [29]. The self-regulation model focuses on a 5-stage self-regulation, including specification of goals, establishment of commitments to change, physical and environmental management to facilitate pursuit of goals, and execution of self-regulation components to achieve the goal [30]. The strength model of self-control consists of the following components: standard of desirable behavior, motivation to satisfy standards, monitoring of situations that achieve the standards, and internal strength to control urges [31]. Self-control is a central function of an individual. This function is an important key to succeed in life [31] because it alters the individual’s responses. In particular, self-control aligns an individual with standards and supports the pursuit of a long-term goal. The conceptual framework (as shown in Figure 1) illustrates the relationships between mHELP and health outcomes.

In this conceptual model, self-regulation involves self-awareness of the current overweight and obese status. Awareness can trigger a self-evaluation response, which involves the interpretation of the condition of an individual against a goal or a standard. In addition, a series of responses can be determined after self-evaluation as a result of self-adjustment and self-reinforcement [30] to improve healthy diet pattern, increase physical activities, and obtain appropriate GWG. Consequently, women who participated in using the mHELP app will probably obtain improved maternal and neonatal outcomes.

### Step 2: Evidence From Our Systematic Review and Meta-Analysis

To obtain the most significant available evidence to design mHELP, our research team conducted a systematic review and meta-analysis to evaluate the effectiveness of electronic-based (e-based) lifestyle interventions in overweight or obese perinatal women [32]. Seven electronic databases, including the Cumulative Index to Nursing and Allied Health Literature, Cochrane Library, Excerpta Medica database, ProQuest Dissertations and Theses, PsycINFO, PubMed, and Scopus, were searched from their inception to July 13, 2016. We selected 14 randomized controlled trials (RCT) in 17 publications among the 1145 available studies [32]. Our review found that e-based lifestyle intervention is an acceptable approach to limit GWG, lose postnatal weight, increase the moderate and vigorous physical activity, and reduce the calorie intake after intervention [32].

Different e-based delivery formats were observed in 14 selected RCTs; these formats include app [12], website [33], the internet [34], email [35], short message services [36], computers [37], and interactive videos [38]. Physical activity, diet, and weight management are essential components in designing healthy lifestyle interventions [32]. Promising strategies play key roles in promoting healthy lifestyle. These strategies include setting of behavioral goals, undergoing lifestyle counseling or skills training, regular self-monitoring, and receiving reinforcement through feedback from health care professionals. Intervention starts in the first trimester of pregnancy until postpartum periods to broaden the beneficial effect of intervention [39]. E-based platforms incorporating in-person and phone session for professional consultation are effective in reducing GWG [32] because synchronous interpersonal interactions may be beneficial in improving the effectiveness of the intervention [40]. Social networking among peer support is vital in promoting healthy behavior [41]. Monitoring device for physical activity [42], image-assisted dietary assessment [43], and anthropometric measures for nutritional status [44] are considered accurate outcome measurements. Table 1 summarizes the suggested recommendations from our systematic review [32]. These recommendations can guide our study in exploring the subsequent step in designing a lifestyle program for overweight and obese perinatal women.

### Step 3: Use of Qualitative Research to Inform User-Centered Design

A qualitative methodology involving end users in the design process was used [25]. Qualitative research method is important because it provides useful insights for initial elicitation to design and develop mHELP [45]. A user-centered approach helps explore the needs and preferences of content, platform, and format of mHELP among overweight or obese multiethnic perinatal women [25]. The outcomes of this qualitative research may facilitate in tailoring the intervention, thereby increasing its acceptability and effectiveness.

#### Sample and Setting

We purposively recruited 16 participants among multiethnic overweight and obese pregnant women in 2 outpatient clinics in a hospital, which is a 1160-bed, university-affiliated hospital that serves more than 670,000 outpatients and 49,000 inpatients. This hospital provides comprehensive obstetric care for different demographic and socioeconomic groups in Singapore, with a delivery rate of 3233 deliveries/year. The inclusion criteria for participating in the study included pregnant women aged ≥21 years with prepregnancy BMI of ≥25.0 kg/m^2^ and having a singleton uncomplicated pregnancy at no more than 30 weeks of gestation.

#### Data Collection

Data collection was conducted from July 2016 to January 2017 after obtaining approval from the Domain Specific Review Board (Reference No: NHG DSRB 2016/00654). A research assistant approached the target pregnant women during antenatal clinic visits, and eligibility screening was performed in a private area. Participants were informed about the purpose of the study, and information sheet was given. This initial contact was followed up by a telephone interview to establish their interest and consent to participate. Informed written consent was obtained, and participants’ profiles were collected. A qualified research assistant, who was trained to conduct qualitative face-to-face interviews, carried out all individual interviews to ensure a high level of consistency. A semistructured interview guide (Textbox 1) with open-ended questions was used to explore the needs and preferences of participants in terms of the content, platform, and format for a healthy lifestyle intervention.

**Table 1:** Recommendations from the systematic review and meta-analysis.

Aspect	Recommendations
Component	Physical activity, diet, and weight management
Period	First trimester to postnatal period
Platform	E-based platform incorporating in-person and phone session
Strategies	Setting behavioral goals, undergoing lifestyle counseling or skill training, regular self-monitoring, and receiving reinforcement through feedback
Outcome measures	Monitoring device for physical activity, image-assisted dietary assessment for dietary intake, and anthropometric measures for nutritional status
Interactivity	Online peer discussion forum, texting, email, or phone for professional consultation
Functionality	Graphs for progress report, navigation, search feature, goal tracking, notification or reminder, and link to remote device

**Semistructured interview guide for face-to-face individual interview.**

What do you think about the healthy lifestyle intervention for perinatal women?

What is the most important content you need to know in a healthy lifestyle intervention?

Do you have any other suggestion regarding the specific contents for a healthy lifestyle intervention?

How do you feel in using electronic technology to deliver healthy lifestyle intervention?

What are your experiences in accessing information from electronic-based platform?

Tell me about how you choose preferable technology platform to receive healthy lifestyle information and why?

What do you think about the favored way to receive information about healthy lifestyle?

What do you think about the favored format for interactivity with peers?

What format do you prefer in communicating with health care professionals?

What is your preferred presentation format using multimedia?

What are the essential aspects of design that will engage you in electronic-based intervention?

Do you have any additional thought that you have not expressed in the presented questions?

Finally, is it alright to contact you for some follow-up questions if necessary?

This interview technique allowed participants to express their answers freely in their own words. The interview questions and prompt were developed according to a framework for a qualitative semistructured interview guide [27]. All interviews were conducted in a private room at the convenience of the participant, either before or after the scheduled follow-up at 2 outpatient clinics. The interview lasted for 20 to 40 min. All interviews were audio-recorded, and field notes were taken. We provided each participant $20 (Singapore dollar) as a token of appreciation for their time.

#### Data Analysis

Descriptive statistics summarized the participants’ characteristics. Data were audio-recorded and transcribed verbatim. We used thematic analysis following the methods of Braun and Clarke [46]. This method was selected because of its flexibility, freedom from specific theoretical framework, ability to explore a rich set of data, and identification and analysis of repeated themes [46]. This analytic process is a theoretically flexible method with 6 recommended steps [46], including (1) familiarization with data by reading and rereading the transcripts, (2) initial coding by systematically identifying and naming units of meaning with codes, (3) searching for themes among the initial codes according to data patterns, (4) reviewing themes by organizing the data that may best fit together as subthemes, (5) defining and naming final major themes, (6) producing the report. The themes identified on a semantic level were closely linked to the data using an inductive approach. Constant comparative analysis was performed to iterate the variation between theme occurrences across different participants [47]. Thematic saturation was achieved at the 16th interview as determined by 2 research team members (YL and LJC) during concurrent analysis. Illustrative verbatim quotations were selected to support the validity of data generation.

#### Methodological Rigor

Credibility, dependability, confirmability, and transferability establish the methodological rigor [48]. To ensure the credibility of data sources, prolonged engagement and member checks were used. We repeatedly viewed the audio recording, transcription, and field notes for accuracy. We sent the transcriptions to the participants to validate the information through the member-checking procedure [49]. Dependability was achieved by auditing. Audit trails were developed using reflexive memos and codebooks throughout the research process to keep track of any bias and assumption [50]. For confirmability, we involved coresearchers in the analysis. Two research members (YL and LJC) independently participated in a multiphase process, including initial coding, theme development, review, and definition [46]. Two members verified this process for accuracy and appropriateness. Any discrepancy was discussed and resolved for consensual validation. Our research team facilitated transferability by providing details of demographic and obstetric descriptions and using relevant quotations from different participants [48].

## Results

### Overview

We invited 20 eligible women, but 4 women refused to join because of lack of time and planning to deliver in another hospital. A total of 16 eligible women (response rate=80%) agreed to participate; of these, 8 were recruited from a general outpatient clinic, and 8 were from a private outpatient clinic. Table 2 presents a summary of the demographics and obstetric characteristics of all participants. The participants’ BMI ranged between 25 kg/m^2^ and 38 kg/m^2^. Majority of the participants were married and with complete tertiary education.

After a 6-step thematic analysis, 5 key themes related to the domains of content, platform, interactivity, format, and functionality emerged. These 5 themes captured the meaning of narratives offering contextual insights into the development of mHELP. Summaries of the key themes, subthemes, and recommendation are presented in Table 3.

**Table 2:** Characteristics of interview participants (N=16).

Characteristics		n (%)
**Ethnicity**		
	Chinese	3 (19)
	Malay	6 (37)
	Indian	4 (25)
	Burmese	1 (6)
	Hispanic	1 (6)
	Bangladeshi	1 (6)
**Age, in years**		
	25-34	11 (69)
	35-45	5 (31)
**Marital status**		
	Married	15 (94)
	Divorced	1 (6)
**Education**		
	Degree and above	11 (69)
	Diploma and below	5 (31)
**Employment status**		
	Full time or part time	11 (69)
	Unemployed or housewife	5 (31)
**Body mass index, in kg/m^2^**		
	25-30 (overweight)	10 (62)
	31-38 (obese)	6 (38)
**Number of pregnancies**		
	1	7 (44)
	2-4	9 (56)
**Number of babies**		
	0	7 (44)
	1	5 (31)
	2-3	4 (25)

### Theme 1: Content

#### Subtheme 1.1: Culturally Tailored and Specific to Pregnancy

Most participants said that they were highly attracted to engage in the intervention if it can provide them with a culture-specific diet plan. Some participants expressed their opinions as follows:

Maybe something related to like different culture. Because I am Malay…maybe the meal plan should be customized to race or culture so that I do not need to adapt diet from other culture.

Participant 7

Whatever I can search pregnant-related information from the internet is mostly developed by the western countries…so the baby size, the body weight of the baby and mother is very much westernized. So I think maybe I should cater to Asian mother and the baby.

Participant 3

Some participants wanted pregnant-specific healthy lifestyle. They stated:

I like all provided information is pregnancy-based, any suggested activities should be pregnancy-friendly.

Participant 1

We are pregnant so it is slightly different from the normal people…so I think information will be very helpful only for the pregnant women.

Participant 3

**Table 3:** Key themes, subthemes, and recommendations. SMS: short message service.

Themes		Recommendation
**Content**		
	Culturally tailored and specific to pregnancy	Culture-specific, pregnancy-related, and credible educational support for diet, exercise, and weight advices for perinatal women
	Multicomponent	
	Credibility	
**Platform**		
	Use of mobile app	Mobile app is used in a user-friendly design
	Convenient and user-friendly	
**Interactivity**		
	Flexible communication with health care professionals	Professional support via email, SMS text messaging, and hotline for individual consultation
	Importance and value of peer support	Peer sharing and support using online peer discussion forum
**Format**		
	Aesthetic appeal	Interactive multimedia, including video, animations, game, and Web-based quiz
	Visualized features	
	Interactive multimedia	
		External link for further resources, such as websites, text, news, and articles
**Functionality**		
	Self-monitoring for individualized goal setting	Graphs for progress report using tracking and notification
	Progress monitoring	Incorporated with remote device (pedometer)
	Regular update	Easy navigation and automatic update

#### Subtheme 1.2: Multicomponent Content

The participants also suggested that multicomponent content possesses a key role in designing the content of intervention according to different educational needs. Intervention components should highly emphasize on appropriate exercises, dietary advices, and weight management. Participants’ preference is illustrated in the following statements:

Usually it’s very helpful to know like which type of food, how many calories or what kinds of food I can eat for controlling my weight.

Participant 5

I actually don’t know to what extend I can exercise. What is suitable exercise for different period of pregnancy?...How much exercise per day or per week that I should do that is good for my body weight?

Participant 7

#### Subtheme 1.3: Credibility

Participants raised the issue about the intervention’s credibility. They felt comfortable with obtaining information from reputable sources, such as their doctors, nurses, university, or hospital, rather than from unknown sources regarding the authorship or institution of origin. Two participants shared their experiences:

Some websites do not provide any information about themselves and I don’t believe it. I like information from doctors or nurses that I can trust.

Participant 7

I think if information source comes from hospital or university, accuracy is the first thing I would expect and credibility is already there.

Participant 8

### Theme 2: Platform

#### Subtheme 2.1: Use of Mobile App

Most participants expressed that the use of mobile app is a preferable technology platform. The main advantage of using mobile apps is that it is handy and easy to navigate; it can be accessed anywhere at any time. Participants stated the following:

Personally, I tried out a number of pregnancy apps. I found mobile phone is quite handy and mobile app is easy to download.

Participant 4

Nowadays, everybody uses mobile phone and everything is on the app. App likes a one-stop place for everyone and I can access anywhere at any time. App can provide hyperlinks that I could click on if I wanted to.

Participant 12

#### Subtheme 2.2: Convenient and User-Friendly

The participants mentioned that the platform should be convenient and user-friendly. They stated:

For me, user-friendly is very important because I like a simple, user-friendly and direct way to see things. I want to go straight down the page and get where I want to go.

Participant 8

User-friendly is good! That we can easy to use to read, key in, and find things.

Participant 14

### Theme 3: Interactivity

#### Subtheme 3.1: Flexible Communication With Health Care Professionals

Participants agreed that Web-based consultation is crucial to provide helpful advice and suggestion. Participants also tend to interact with health care professionals in a flexible manner through different delivery modes. Participants stated:

I want some experts provide professional advices on appropriate exercise and balanced diet so online consultation should be very helpful.

Participant 11

I might not be able to make the interaction in person because of transportation issue or I might have my work. It’s easier for me to consult them through phone, email or Skype if there is any misunderstanding I can ask for clarifications.

Participant 7

#### Subtheme 3.2: Importance and Value of Peer Support

Participants felt that intervention can potentially provide professional support and emphasized the importance of peer support from online discussion forums. They stated:

It is a good to have our online discussion forums with other pregnant women and we can exchange our views or maybe even get some information and some help through online discussion forum.

Participant 2

I enjoy online chatting with other pregnant women and it can offer tips and suggestions for my pregnancy. Honestly, I want reassurance about my status during pregnancy and it means a lot to me if someone’s back up.

Participant 11

### Theme 4: Format

#### Subtheme 4.1: Aesthetic Appeal

Most of the participants pointed out that the aesthetic appeal of the intervention is a major determinant in their usage of the app. Thus, considering a colorful and attractive interface is essential. Two typical answers are shown below:

Of course bright color as it can attract people to look at it…so it is easy to catch attention and facilitate learning.

Participant 3

I prefer colorful design such as pastel color. Generally, I relate pastel color to baby. Pink for girls and baby blue for boy.

Participant 4

#### Subtheme 4.2: Visualized Features

Visual information regarding the quantity and type of food to eat during pregnancy is also an important consideration. Visualized features improve the ability of women to grasp their progress and recognize their food intake. Participants commented:

I like more visuals and graphics for knowing my condition.

Participant 3

It is really good to show me what a standard serving size is supposed to look like because I want to see it.

Participant 10

#### Subtheme 4.3: Interactive Multimedia

The general preference of the participants is the use of interactive multimedia, including short video scripts, graphs, photos, Web-based quizzes, and animation, to make the app highly engaging. Some participants commented that they appreciate the option of using different multimedia to engage in the intervention, as echoed in the quotes below:

Short video for suggested exercise, health tips and the recommended diet would be interesting and useful.

Participant 6

Personally, I prefer more photos, animation and graphics. I feel online quiz is an awesome part. Once I read the information, then I just check whether I have understood it correctly through quizzes.

Participant 8

### Theme 5: Functionality

#### Subtheme 5.1: Self-Monitoring for Individualized Goal Setting

The participants recognized that each pregnancy presents distinct challenges. They want content in terms of healthy lifestyle tailored to their needs and expectations. A general consensus by the respondents indicated that the program should possess functions that will allow self-monitoring. Two participants shared their ideas as follows:

I would like to have some graphs for monitoring my diet, activities and weight that is good.

Participant 7

I can insert my personal information for the doctor to see using the graph or the chart to monitor how well do I manage, the things to do prior to the next appointment so that the doctor can review.

Participant 10

#### Subtheme 5.2: Monitoring the Progress

Participants felt that the contents should motivate and remind the users of their progress. The participants expressed a desire to monitor their food intake, physical activities, and weight. This result corresponded to other statements from other participants, as evidenced by the quotes below:

I need to motivate and remind myself. It is really good if the intervention can give me notification to keep on track.

Participant 4

Hoping to have simple diet, weight and exercise device or tool to monitor my progression and a routine pop-up message is helpful for reminding me.

Participant 14

#### Subtheme 5.3: Regular Update

Given the rapid change in knowledge, regular updating of content is considerably important. Participants requested for a regular update on the app as a part of the functionality of the intervention. Two participants commented as follows:

I like to read current research and know the latest perinatal diet advice or suggested exercise.

Participant 7

It will be good if weekly update from intervention to get more updated knowledge.

Participant 8

### Translating Theory, Evidence, and User Needs Into Intervention

On the basis of the results from the theoretical basis and recommendations of our systematic review and qualitative research findings with target users, the program content, platform, and format of mHELP were formulated. A multidisciplinary research team was formed. This team included a computing expert, a professional app designer, an obstetrician, a dietician, a physiotherapist, a nursing specialist, and a researcher to design mHELP. mHELP aims to improve the neonatal and maternal outcomes in overweight or obese multiethnic perinatal women. To design a highly automated mobile app with a user-friendly interface, mHELP’s user interface and features were designed by a computer expert and a professional app designer. The app can be preinstalled on phones and delivered as a Web app to provide online colorful features, such as social networking and tracking within a Web browser. The software program was used in multiple mobile platforms, such as Android and iOS (iPad and iPhone). A visual graphing function was designed to allow the user to configure their starting weight, food intake, and physical activity throughout the pregnancy. Real-time feedback and automatic notification were also developed in the app. Our research team will incorporate social functionality in a large, open-source software hosting service GitHub that makes a developer’s identity, so that mHELP can be publicly visible across a wide community.

**Figure 2:**
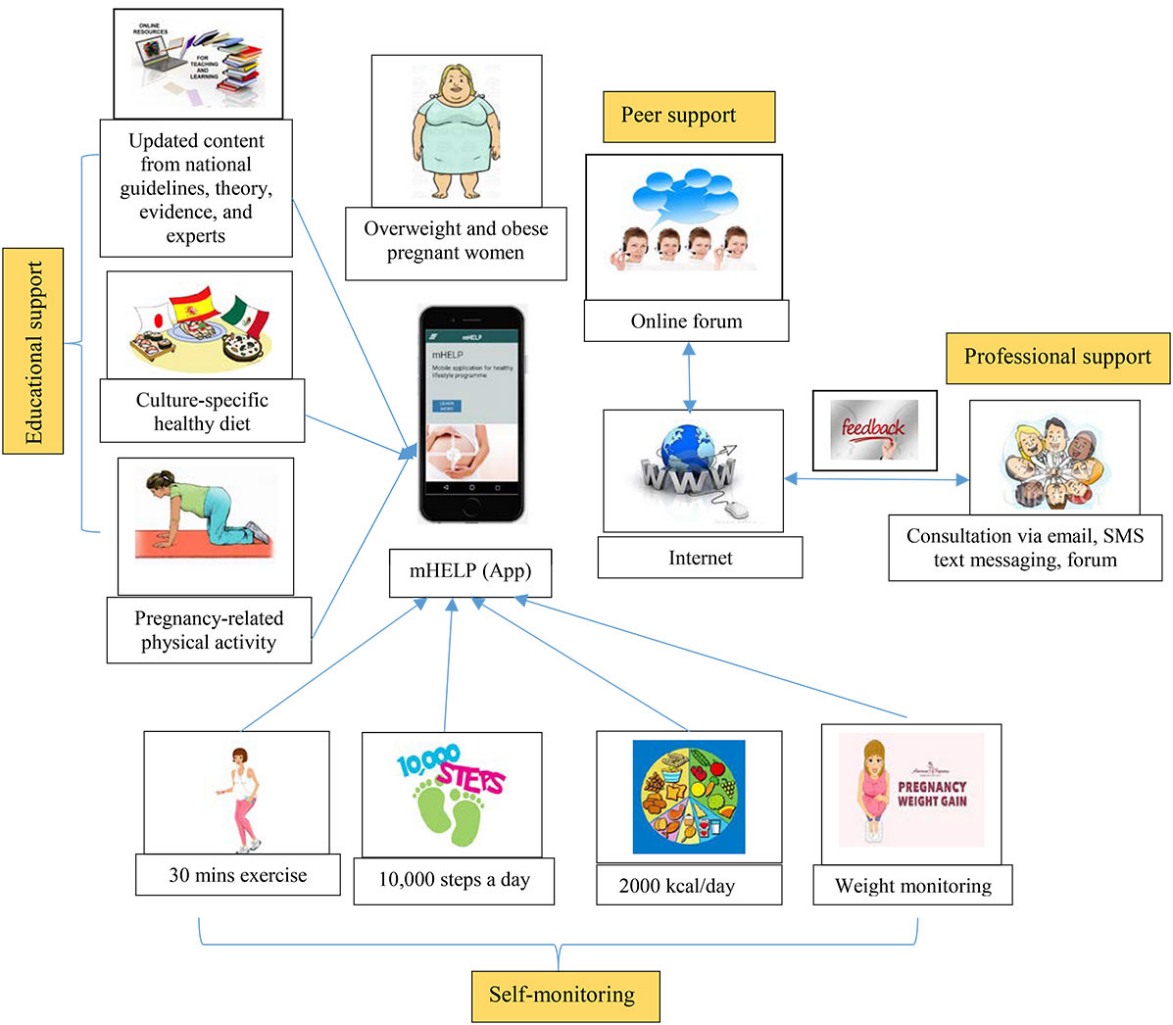
User story box of the mHELP.

**Figure 3:**
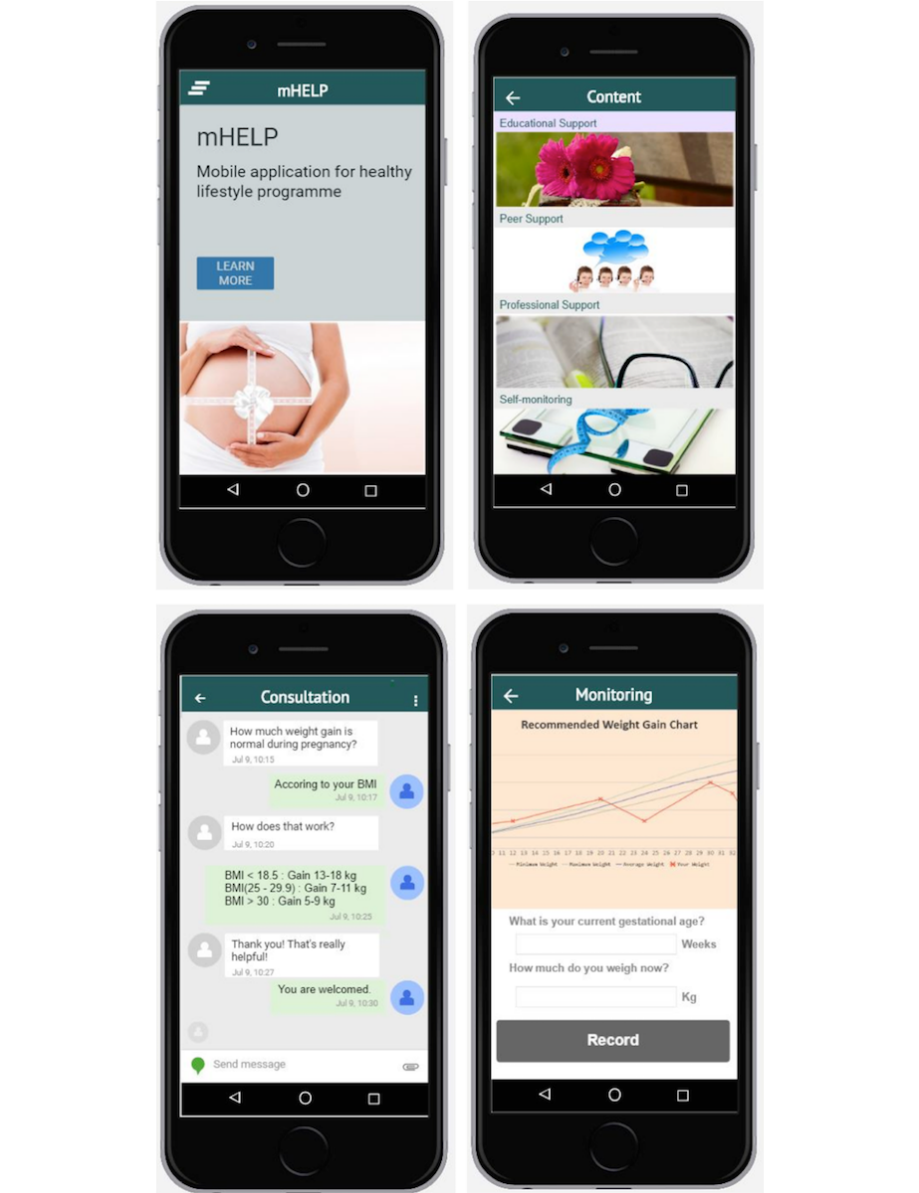
Mock-up screenshots of mHELP.

mHELP content consists of healthy diet advice, appropriate physical exercise, and weight management starting from 12 weeks of gestation to 6 months postpartum, as shown in Textbox 2. Users can access mHELP in their own time and at their own pace. They can also revisit the contents without time limitation. This app contains culture-specific, pregnancy-related, and credible contents, including educational, professional and peer support, and self-monitoring domains. Educational support aims to motivate participants by determining the importance of changing their diet and physical activity to maintain appropriate weight gain. Peer support aims to mediate interaction of participants with one another using a pseudonym via online peer-to-peer communities, which are used to mobilize and raise collective awareness [51]. Professional support aims to achieve adherence and enhance healthy lifestyle knowledge through asynchronous and synchronous feedback [52]. Self-monitoring aims to encourage users to self-regulate their lifestyle behavior according to their goals and reinforce any change made. If the result is below or above the range, then the system provides a notification through the app. The user story box and mock-up screenshots of mHELP are illustrated in Figures 2 and 3, respectively, and its description is presented in Textbox 2.

**mHELP description.**

- **Technology platform**: mHELP is a responsive Web app that can be used on personal computers, tablets, or smartphone devices through common Web browsers and operating systems
- **User interface**: colorful and user-friendly
- **Content**: multicomponent with 4 domains

**Domain 1: Educational support**

1. Content
  - Content is obtained from national guidelines, theory, evidence, and experts.
  - Information is updated and contents are constantly updated to maintain the user’s interest
  - Mobile app incorporating in-person communication via Skype, email, short message services (SMS), and phone session
2. Pregnancy-related physical activity
  - Safety issue for physical activity during perinatal period
  - Aerobic exercise: (1) walking, (2) swimming, and (3) yoga
  - Strength training: (1) foot and ankle exercise, (2) calf stretch, (3) pelvic floor exercise, and (4) pelvic tilting
3. Culture-specific healthy diet
  - Appropriated weight gain
  - Healthy diet plan in Chinese, Indian, and Malay styles
  - Additional nutrient needs during pregnancy
  - Pregnancy food myths
4. Format and security
  - Interactive multimedia, including short video scripts, animations, illustrations, games, and online quizzes
  - External link to credible resources
  - Customizable details and user settings
  - Password-protected to ensure online security
  - User portal via a privacy compliant–shared record platform

**Domain 2: Professional support**

1. Content
  - Feedback from health care professionals (obstetrician, dietician, physiotherapist, and nurse) will improve the compliance and knowledge.
2. Interactivity
  - Online forum for group consultation
  - Users can contact through email, SMS, and/or telephone for individual advice from experts if needed
  - Content-related questions are addressed promptly

**Domain 3: Peer support**

1. Content
  - Provide a forum for the participants to converse with one another
2. Interactivity
  - Online peer-directed forum for sharing and support
  - User can see how others responded to the poll questions about healthy lifestyle
  - User can read posts by other participants; they can also contribute responses in the online forum

**Domain 4: Self-monitoring**

1. Individualized goal setting
  - Physical activity: engaging in 30 min of moderate to vigorous physical activity at least 5 days per week or walking 10,000 steps a day
  - Dietary: improving or maintaining the nutritional quality of their diets by consuming 5 servings of fruits and vegetables per day based on the 2000 kcal/day requirement and avoiding excess sugar and fat intake and emotional eating
  - Weight: following Institute of Medicine–recommended weight gain during pregnancy
2. Functionality
  - Users set individualized behavioral goals, and goal achievement triggers onscreen congratulatory feedback. Generate a list of goals in the form of action plan per week and archived goal content
  - Offer visualization tools, such as graphic progress chart and food image–assisted dietary advice or assessment
  - Navigation, tracking, and notification
  - Synchronized with remote monitoring devices (pedometer)
  - Tailored messages displayed on app and delivered via SMS or email according to the user’s preference
  - Regularly updating app with current content
  - Auto-update function for app system

## Discussion

### Overview

To our knowledge, this study is the first to use a theory-informed, evidence-driven approach, and user involvement in developing a mobile app for a healthy lifestyle program in overweight or obese multiethnic perinatal women. We provide the description of a 3-step process by integrating theoretical basis, evidence from our systematic review, and research findings from target users. In addition, multidisciplinary research team was formed to provide expert advice and contribute practical and clinical considerations in designing mHELP.

### Principal Findings

We developed a theory-based intervention following the TIDieR intervention guideline [20]. Integrated concepts from social cognitive theory of self-regulation [29], self-regulation model [30], and strength model of self-control [31] were used to provide theoretical guidance on the app’s development. Our systematic review and meta-analysis synthesized the most remarkable evidence using 14 RCTs [32] to provide valuable recommendations on component, period, platform, strategies, outcome measures, interactivity, and functionality of the intervention. With consideration that meta-analysis is the strongest and highest quality of evidence [24], mHELP suggested an evidence-based design. Furthermore, our qualitative study among potential users builds on our previous systematic review and meta-analysis [32] to explore the needs and preferences to design a tailored intervention. The use of a qualitative approach to elicit the user’s views during intervention development is considered a good practice [45]. This user-centered design is tailor-made to the end-user perspectives because it can ensure that the app is desirable and suitable for end users [26] by selecting preferable content, technology platform, user interface, interactivity, and functionality.

Themes that emerged from our qualitative study suggested that culturally tailored, pregnancy-specific, multifaceted, and credible contents are particularly important. Given the multiethnic groups in Singapore, we considered to make the content culturally sensitive by matching health information to the observable characteristics of a target group. This effort involves translations to Chinese, Malay, and Indian using pictures of pregnant women with various ethnic backgrounds and using food items familiar to and preferred by pregnant women in those cultures. Users are likely to engage actively in mHELP if they perceive that the intervention is relevant to pregnancy. Multifaceted contents provide a first exposure to educational information in virtual learning environment to satisfy educational needs. Educational support may motivate active learning; in addition, the overall concept is that the users take ownership of their learning [53]. Design format should include aesthetic appeal, visualized features, and interactive multimedia. Multimedia features can accommodate different learning styles [54]. Data visualization improves the user’s ability to comprehend their progress and monitor their food intake [43]. Contents should provide realistic goals and practical strategies to initiate change in the target behaviors [18].

Among a range of technology platforms, our mobile app satisfies the need for ubiquitous technology resulting from our qualitative study because of its convenient and user-friendly interface. The widespread use of mobile technology, along with the availability of efficient mobile broadband connections, offers a distinct opportunity to develop an innovative learning method [55]. Users can learn about healthy diet, appropriate physical activity, and weight management by using short interactive videos, animations, games, or Web-based quizzes [55,56]. Mobile app has gained popularity among perinatal women [57] because of its handy features, easy usage, and multifunctional attributes. Mobile apps also offer self-regulatory features, which may promote personal awareness of health behaviors in users [13,14]. With regard to interactivity, users preferred flexible communication with health care professionals through the provision of Web-based discussion and access to individualized expert advice. Cloud computing offers flexible dissemination channels between health settings and health care providers [58]. Peer support is an important element of the intervention, which allows users to share their experiences, knowledge, and emotional, social, or practical support with one another [51]. Furthermore, we need to update the content and system continually to ensure that the content is updated and credible. Optimizing and maintaining user engagement remain a considerable challenge. Hence, engagement strategies are essential to app designs. These strategies include ease of use, aesthetic design, feedback function, ability to change designs to suit an individual’s preference, tailored information, and distinct mobile phone features [59].

### Implications

We demonstrated that a 3-step approach can be applied to develop mHELP for overweight or obese multiethnic perinatal women. Results are useful to design a culture-specific, multifaceted, and user-friendly app. The ubiquity of app facilitates the dissemination of information, supports a broad range of audience, and allows the tailoring of information and support according to users’ characteristics and experiences [60]. The popularity of technological advancement can indicate a shift toward maternal empowerment within the maternity care provision [14]. Overweight and obese perinatal women can access mHELP at any time and place. Hence, mHELP can provide support for perinatal women between consultation visits, thereby reducing the number of required outpatient clinic visits [61].

### Limitations

Our study presents several limitations. First, the purposive, regional, and small sample in one hospital may limit the generalizability of our findings. Second, the time to develop a 3-step process is long, and time lags may also occur because of the changeable consumer profiles and fast-paced technological development. Third, app intervention development, including time, labor, facility, equipment, and training, is considerably resource-intensive. Thus, policy makers should consider providing financial support, manpower, protected time, and logistic support for app development.

### Future Work

This paper supports the use of a 3-step process as evidence of the usefulness of this approach. We accommodated the perspective of potential user with theoretical basis and evidence for mHELP development. Further work is needed to perform beta test in the feasibility study before RCT. In beta testing, we will evaluate the clarity of language, ease of screen navigation, technical bugs, corrupt hyperlinks, and typographical errors in various internet browsers. In addition, we will conduct a qualitative study to elicit the users’ experiences after intervention. Further application and refinement will help establish evidence about the acceptability, usability, adherence, sustainability, and cost-effectiveness of mHELP. After mHELP refinement, we will evaluate its effectiveness in large and well-designed RCTs in different settings. Hence, mHELP is tailored as a culturally relevant app for obese and overweight perinatal women.

### Conclusions

With the growth of smartphone devices, a series of mobile apps has been developed to provide education, information, and support concerning health problems. Theory, evidence, and user needs are vital in intervention development. The iterative process allows the incorporation of end-user feedback, theories, and systematic reviews to formulate the content, platform, and format of mHELP, which is tailored to the user’s preferences. Our 3-step developmental process is a useful guide for researchers or app developers for future app development.

## Abbreviations

BMI: body mass index
e-based: electronic-based
GWG: gestational weight gain
RCT: randomized controlled trial
SMS: short message service
TIDieR: Template for Intervention Description and Replication
